# Muscular tension in ear surgeons during cochlear implantations: does a new microscope improve musculoskeletal complaints?

**DOI:** 10.1007/s00405-024-08899-0

**Published:** 2024-08-22

**Authors:** Antonia Lakomek, Theda Eichler, Moritz Meyer, Benedikt Höing, Marcel Dudda, Stephan Lang, Diana Arweiler-Harbeck

**Affiliations:** 1 Department of Otorhinolaryngology, Head and Neck Surgery, University Hospital Essen, Hufelandstraße 55, 45147 Essen, Germany; 2 Department of Trauma, Hand, and Reconstructive Surgery, University Hospital Essen, Hufelandstraße 55, 45147 Essen, Germany; 3 Department of Orthopaedics and Trauma Surgery, BG Klinikum Duisburg, Duisburg, Germany

**Keywords:** Cochlear implantations, Muscle tension, Type of microscopes, Work-related disorders

## Abstract

**Purpose:**

Musculoskeletal complaints and fatigue are commonly described symptoms in daily work of ear nose and throat surgeons using a microscope. Long ear surgical procedures are associated with prolonged microscope use, which can lead to unconsciously tense and uncomfortable body posture. The digital microscope RoboticScope^®^ allows visualization of surgical site through a Head-Mounted Display, independent from camera head and is therefore much easier on the back, as the sitting position can be adjusted flexibly. Aim of the prospective study was to investigate to what extent the use of a RoboticScope^®^ changes the tension of neck and shoulder muscles of the surgeons in comparison to a conventional tripod microscope.

**Methods:**

For this purpose, the electric activity of neck and shoulder muscles of surgeons was recorded using surface electromyography during cochlear implantations. Electrical potentials were derived via electrodes on neck and shoulder muscles. The basic tension of those muscles was measured in relaxed position before and after surgery. During microscope use the tension was continued to be measured. A questionnaire recorded parameters such as level of difficulty of operation as well as patient data.

**Results:**

Results from 58 operations, 33 of which were performed using a conventional microscope and 25 using a Head-Mounted Display, show a significant reduction in muscular tension during surgery by 40% (*p* < 0.001) in experienced ear surgeons when using a RoboticScope^®^, regardless of the surgeon.

**Conclusion:**

Our results are in line with the relevance of preventive measures to avoid acute and chronic work-related illnesses/symptoms described in literature.

## Introduction

Musculoskeletal complaints and fatigue are commonly described symptoms during and after work of surgeons who work frequently and for long periods with conventional microscopes [1]. The prevalence of work-related disorders in ear, nose and throat (ENT) surgeons during their working life is reported between 47 and 90% by Story et al. [2]. Especially ENT surgeons are using microscopes, regardless of whether digital or analog, which lead to a stooped, tense body posture, that increases the risk of musculoskeletal disorders [3]. Dahmash et al. showed that particularly the neck and shoulder muscles are affected in ENT doctors [4].

Big national wide studies of several countries showed the impact of muscular discomfort and pain in ENT surgeons [5–7]. In a large study by Vijendren et al. 2016, approximately 50% of the participating ENT physicians in England reported musculoskeletal complaints [5]. Additionally, in a Canadian study, carried out on surgeons who operate with microscopes, 97% of the participants experienced some physical symptoms in one or more than one part of their body [6].

Many more countries describe that work-related musculoskeletal complaints repeatedly lead to work absenteeism, more than other diseases do [8].

There are several studies describing the increasing problem of occupation-related physical complaints in ENT surgeons. However, there are only a few studies on possible approaches towards solutions and improvement of specific conditions to mitigate surgeons’ complaints [2].

Particularly ear surgeons suffer from a poor neck posture, while using a normal tripod microscope. The digital microscope RoboticScope (BHS ^®^) allows visualization of the surgical site through a Head-Mounted Display (HMD), independent of the camera head. The camera head of the microscope can be controlled by tiny movements of the surgeons’s head; thus the hands don’t need to be taken out of the surgical field to adjust the microscope. Since control via a HMD implies a less cramped posture because of a more upright posture especially in the cervical spine and thoracic spine area. This allows the surgeon to adopt a flexible, relaxed and less static posture.

Hence, we investigated the potential benefit in less muscular tension regarding ear surgeons using a HMD instead of tripod microscope.

## Materials and methods

The study presented here is a single-center prospective clinical trial that to our knowledge for the first time investigates the muscle tension of ear surgeons during ear surgery using the example of cochlear implantation procedure.

The cochlear implantation surgeries were either performed with a digital tripod microscope with a conventionally anchored ocular or a digital microscope RoboticScope^®^ with a HMD. There was no randomized preselection of which surgery was performed with the conventional microscope or with the HMD steered one. The choice of microscope depended on the electrode types of the cochlear implant.

Included cochlear implantation surgeries were performed by two experienced ear surgeons (1 female, 1 male). Neither surgeon had any diagnosed musculoskeletal diseases.

The electrical muscle activity of the neck and shoulder muscles of surgeons was recorded using surface electromyography (EMG) during cochlear implantations. The software Biofeedback 2000Xpert ^®^ was used. Electrical potentials were derived at skin level via electrodes on the upper trapezius muscle. The potentials of the right and left muscle components were recorded separately and displayed as a function of time using an external computer (unit of measurement microvolt).

The basic tension of those muscles was measured in relaxed sitting position before and after surgery for one minute. During microscope use the tension was measured continuously. The mean values over time were calculated for each observation phase.

Furthermore, the starting time and the duration of the surgery, the implanted side, age and BMI of the patient has been noted. The BMI evaluations only included adults, as infants and toddlers typically undergo a different calculation [9].

Before surgery, the surgeons were asked to fill out a questionnaire, regarding parameters such physical activity on the previous day, stress level, subjective muscle tension and number of operations already performed during the day. Afterwards the surgeons had to rank the level of subjective difficulty of the surgery. The answers were determined using an analog scale from very low (0) to very high (10) and were summarized for the evaluations into three levels: low (< 3), medium (3–6) and high (> 6) [10].

The evaluations were conducted depending on the used microscope (conventional tripod microscope and digital microscope RoboticScope^®^ with an HMD.

### Statistical analyses

For statistical analysis SciPy (Virtanen 2020) and for visualising the data Seaborn (Waskom 2021) was used. T-tests were performed to calculate the significance for independent samples and the Pearson correlation was used as a measure of correlation between two independent variables.

## Results

58 cochlear implant surgeries were included in the analysis. 25 were performed using the digital microscope RoboticScope^®^ with a HMD and 33 using a conventional tripod microscope.

**Fig. 1:**
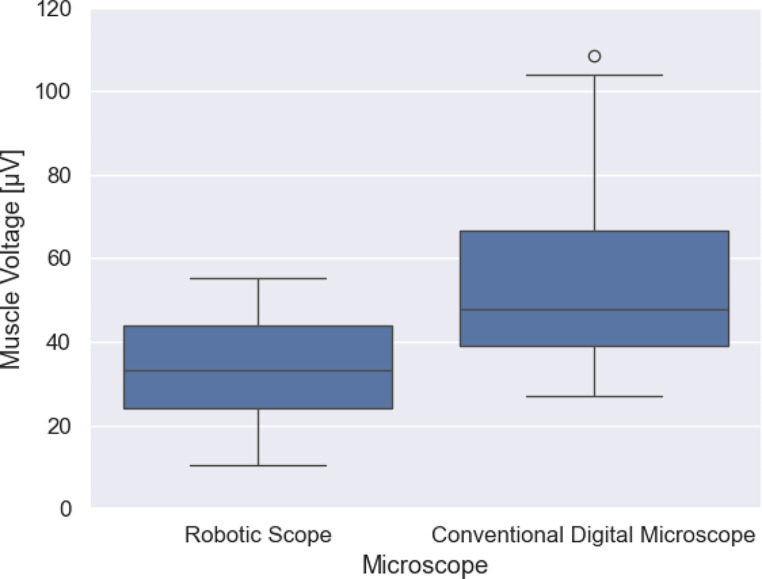
Muscle voltage during surgery: The box plot shows the distribution of the average muscle tension during surgery using the RoboticScope^®^ on the left and using a conventional digital microscope on the right side

A significant reduction by 39.75% of the mean muscle tension during surgery while using the RoboticScope^®^ (RoboticScope^®^ 33.59 µV; *n* = 25 vs. conventional microscope 55.81 µV; *n* = 33; t-test, *p* < 0.001) (Fig. 1) was observed.

With both microscopes, the mean of the resting baseline tension before surgery was higher than after surgery (Table 1). The difference of tension reduction between pre and postoperative was significantly larger with the conventional microscope (*p* = 0.052) (Table 1).

**Table 1:** a) Mean muscle tension pre- and post-surgery in dependence on the type of microscope. b) Body-side-related muscle tension of the surgeon in correlation of side of surgery (side of ear undergoing surgery) independent of the type of microscope

	Both microscopes	RoboticScope^®^ (HMD)	Conventional microscope	*p*-value
a) Mean muscle tension pre- and post-surgery				
Mean muscle tension pre (µV)	30.33 (*n* = 54)	22.06 (*n* = 22)	36.01 (*n* = 32)	0.011
Mean muscle tension post (µV)	24.21 (*n* = 53)	19.24 (*n* = 20)	27.21 (*n* = 33)	0.087
∆ mean muscle tension pre post (µV)	-5.47 (*n* = 52)	-0.94 (*n* = 20)	-8.30 (*n* = 32)	0.052
b) Muscle tension in correlation to the side of surgery				
Right ear surgery – Mean muscle tension right (µV)	46.90 (*n* = 32)			0.096
Right ear surgery – Mean muscle tension left (µV)	36.76 (*n* = 32)			
Left ear surgery – Mean muscle tension right (µV)	55.18 (*n* = 24)			0.135
Left ear surgery – Mean muscle tension left (µV)	44.40 (*n* = 24)			

Furthermore, the tension of the right neck muscles averaged across all surgeries and microscopes was higher than the left regardless of the operated side (Table 1). However, the tension of the right side during surgery of the right ear, was lower than during surgery on the left side (Table 1).

**Fig. 2:**
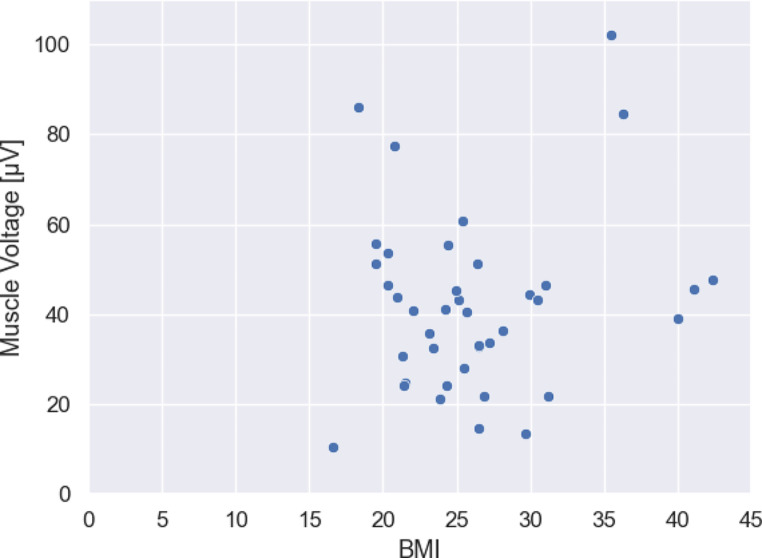
Relation between Body-Mass-Index (BMI) and mean muscle tension: The figure shows the relation between BMI of the patient undergoing surgery and the mean muscle tension during surgery of the surgeon independent of the used microscope

Regarding the Body Mass Index (BMI) of the patient undergoing surgery no correlation was found between higher muscle tension of the surgeon and higher BMI of the patient (*r* = 0.152; *p* = 0.343) (Fig. 2).

**Fig. 3:**
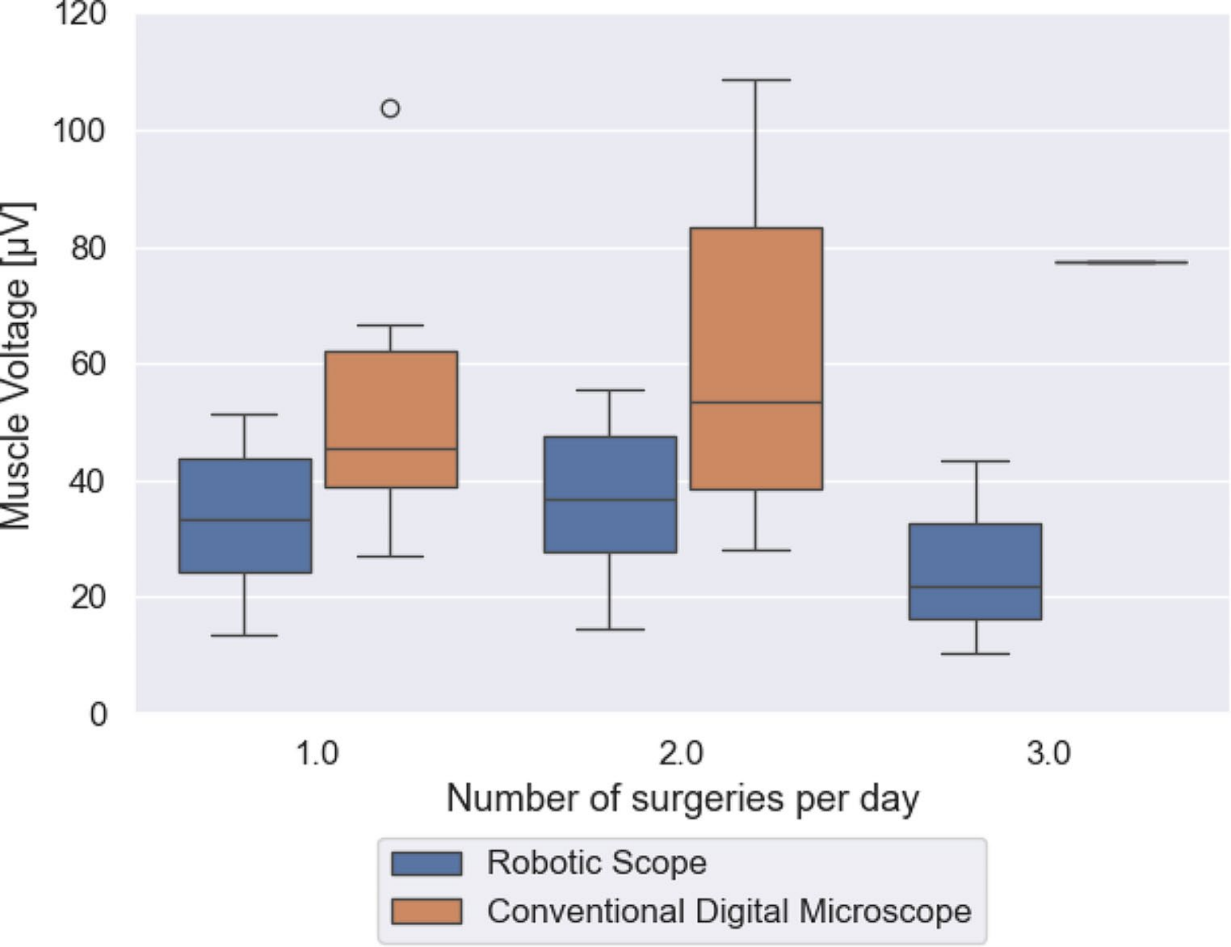
Muscle tension in relation to number of surgeries per day: The figure shows the distribution of the average muscle tension during surgery on the left when the measured surgery was the first of the day for the surgeon, in the middle when it was the second, and on the right when it was the third. Surgeries using the RoboticScope^®^ are represented by the blue plots, and surgeries using the conventional digital microscope are represented by the orange plots

Concerning the number of surgical interventions before the cochlear implant surgery, an increasing muscle tension using the conventional microscope from the first to the second surgery (*p* = 0.303) was noted. While using the Robotic Scope an increased muscle tension only from the first to the second surgery was noted, however it was a lower increase in comparison to the conventional microscope (Fig. 3). It was not investigated whether the preliminary operations were performed with or without a microscope.

With respect to the subjectively perceived difficulty level of the surgery, there was an increase of muscle tension from low to medium difficulty level regardless of the microscope (low difficulty level: RoboticScope^®^ 33.96 µV; *n* = 15 vs. conventional microscope 55.93 µV; *n* = 20; medium difficulty level: RoboticScope^®^ 36.32 µV; *n* = 5 vs. conventional microscope 57.85 µV; *n* = 3; p-value both microscopes low vs. medium *p* = 0.767). There was a trend of a decrease of muscle tension from medium to high difficulty level (high difficulty level: RoboticScope^®^ 27.16 µV; *n* = 2 vs. conventional microscope 32.19 µV; *n* = 1; p-value both microscopes medium vs. high level *p* = 0.047).

**Fig. 4:**
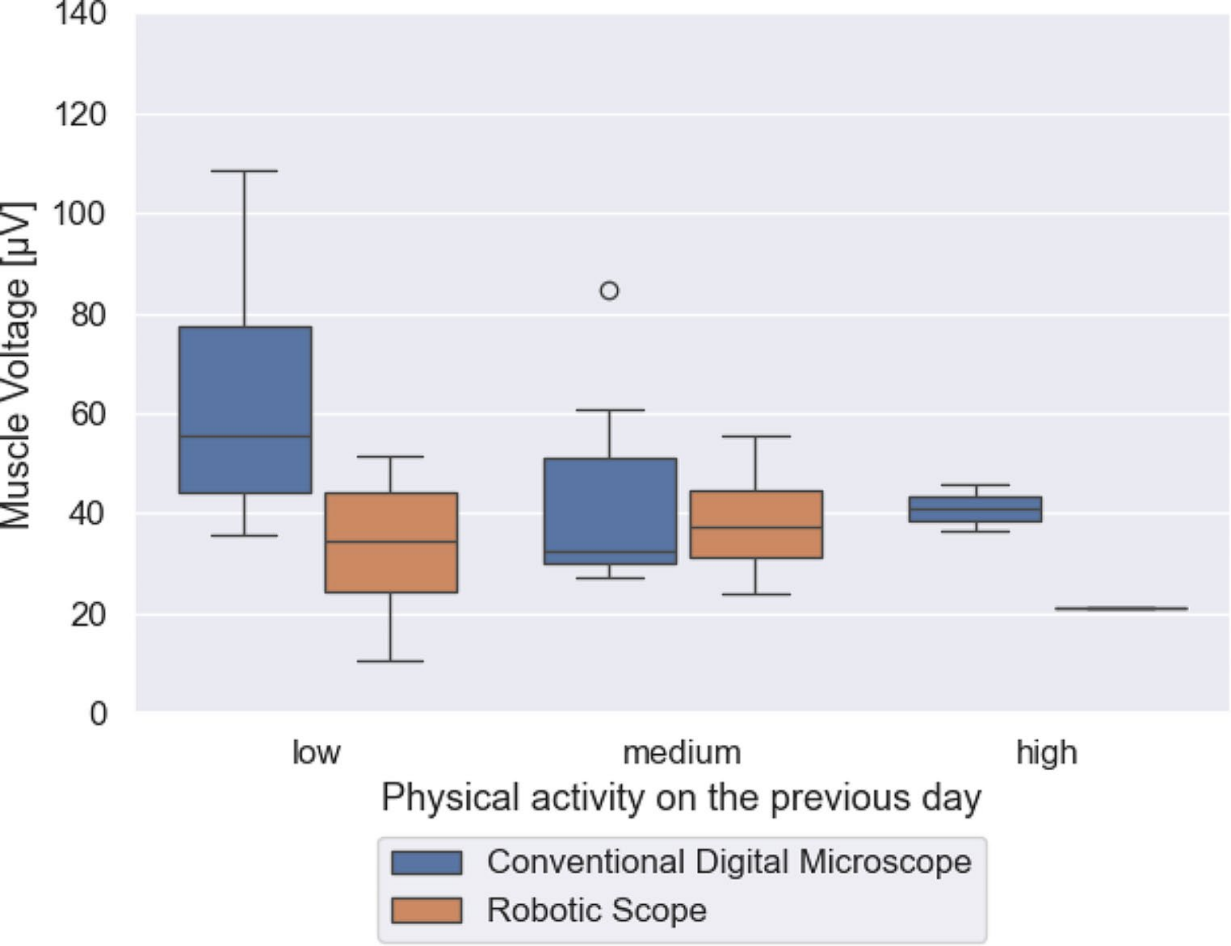
Muscle tension in relation to physical activity on the previous day: The figure shows the distribution of the average muscle tension during surgery on the left when the surgeon had low physical activity the day before, in the middle when the surgeon had medium physical activity the day before and on the right when the surgeon had high physical activity before. Surgeries using the RoboticScope^®^ are represented by the blue plots, and surgeries using the conventional digital microscope are represented by the orange plots

The private physical activity the day before surgery led to a decrease of muscle tension if there was a medium and high physical activity the day before. Regarding the use of RoboticScope^®^ there was in particular a difference between medium and high physical activity (Fig. 4).

The influence of subjective stress levels did not show a meaningful correlation with the measured muscle tension.

## Discussion

The presented study shows the everyday unadorned muscular tension experienced by an ear surgeon during highly demanding cochlear implantations while using a conventional tripod microscope. In line with other studies, such surgeries could lead into a head-bent and back-bent position with resulting in work-related musculoskeletal disorders [7].

A new technology of using a HMD for steering the camera head, now provides the opportunity for an upright, no longer stooped posture. It could be shown that there is a reduction of 40% of muscle tension while using a RoboticScope^®^, expected caused due to the upright relaxed body position.

Interestingly, there was a higher muscle tension in resting position before starting surgery in comparison to the post-surgery measurement. This observation may hint, that a certain mental tension, before a potentially challenging surgery, can lead to higher muscle tension. However, there was a significant bigger difference from pre- to post-surgery measurement while using the conventional microscope.

All surgeons were right-handed. In line with this there was higher muscle tension on the right upper trapezius muscle independent of the sight of surgery and independent of the used microscope. Nevertheless, while performing surgery on the right ear there was seen a lower muscle tension. This may be driven by the possibility for resting the right arm on the operating table while operating the right ear.

It could therefore be questioned, if a storage option for the right arm should be arranged during surgery on the left ear, to also reduce the muscle tension of the surgeon independent of the microscope. This assumption is supported by the findings of Vijendren et al. 2019, who showed a reduction in muscular complaints in microscope based surgery while using a special chair, which holds the operator’s head and provides armrests [11].

With a higher BMI, the surgeon’s sitting position could be impaired, as he or she sits further away from the patient due to the abdomen and arm and would therefore have to operate from a more outstretched arm. Surprisingly, there was no correlation between the BMI of the patient and the muscle tension of the surgeon. This could be caused due to the condition, that not the tissue overall but the degree of pneumatisation of mastoid bone has an influence of the effort of the surgery. The pneumatisation of mastoid bone depends on the development in childhood and has no primary relation to weight [12], therefore no connection between BMI and pneumatisation can be established.

As far as observed, there was an increase in muscle tension associated with the number of surgeries already performed by the surgeon on that day. While using the RoboticScope^®^ there was only an increase from the first to the second surgery, which was much lower than the increase using the conventional microscope. Hence, it seems that the muscle tension caused by the bent over body position while using a conventional microscope intensifies over the course of the day and across the number of surgeries. This is in line with Schechet et al., who showed on the example of ophtalmologists, that surgeons who are doing a lot of surgeries and spending a lot of time in the operating theatre are at risk for musculoskeletal disorders [13].

Regarding subjective parameters of the performed surgery the perceived difficulty level of the surgery has an influence of the muscle tension regardless of the microscope. More demanding surgeries lead into a higher muscle tension. However, from the second to the third surgery a decrease was noted, but it needs to be mentioned that only three surgeries were classified as high level, so that it might be difficult to make a reliable statement here.

An intriguing observation of the study reveals that especially when using the conventional microscope, high physical activity of the surgeon on the previous day leads to a reduction in muscle tension during the operation. This appears to be an important finding. Naturally, we were able to clearly demonstrate that new microscope techniques such as the RoboticScope^®^ lead to a significant physiological improvement in the conditions of the operation. One could also venture to hypothesize that more relaxed operating conditions lead to better surgical outcomes. However, the conventional microscope is not likely to disappear from the everyday life of an ENT doctor in the near future, so it is necessary to consider in which way the conditions at the conventional microscope can be improved. Promoting physical activity in everyday life improves the conditions of the surgeon in their profession. In Germany, it is common practice in many companies to improve the physical activity and fitness of the employees. However, this is by no means commonplace in hospitals. Therefore, it is important to conduct studies that identify and highlight possible improvements in working conditions with the conventional microscope.

### Strengths and limitations

The work underlies the typical constraints of a prospective clinical single-center study based on a small study population. Even though 58 cochlear implant surgeries in a few months in a single-center are a lot, statistically significant statements are difficult to make. In addition we only conducted the study with two experienced surgeons with most expertise to exclude a bias.

Results of the presented study could be regarded as hypothesis generating and more studies should be conducted. Whether the reduction in tension on the back has a long-term effect and leads to fewer back problems cannot be answered from the study. In the study, we were able to measure the maximum and minimum tension as well as the mean tension during the operation. The respective parameters are subject to fluctuations and are susceptible to artifacts.

## Conclusion

In this single-center prospective study setting we could for the first time objectively show, that using new microscope technologies like a HMD separated from the camera body improve the work-related muscle tension in ear surgeons. We identified the performance of multiple surgeries in succession and low physical activity of the surgeon as risk factors for a strong muscular tension during surgery.
